# Secondary angle closure glaucoma due to iatrogenic big bubble formation in manual DALK for stromal degeneration: An unusual presentation

**DOI:** 10.22336/rjo.2023.50

**Published:** 2023

**Authors:** Shreesha Kumar Kodavoor, Pranessh Ravi

**Affiliations:** * Department of Cornea and Refractive Services, The Eye Foundation, Coimbatore, Tamil Nadu, India

**Keywords:** DALK, big bubble, secondary angle closure glaucoma, spheroidal degeneration, surgical management

## Abstract

A 65-year-old male was planned for Deep anterior lamellar keratoplasty (DALK) to improve his visual quality from the underlying spheroidal degeneration along the visual axis. An attempt for a big bubble was futile, hence converted to a manual DALK. On postoperative day 1, he developed secondary angle closure glaucoma due to reverse pupillary block by the host DM. Decompression of the big bubble was done and angle closure was resolved with a good visual outcome at 1-month post-op.

We present the first case of secondary angle closure glaucoma due to reverse pupillary block by a big bubble in a rigid cornea.

**Abbreviations: **DALK = Deep anterior lamellar keratoplasty, OD = oculus dexter, OS = oculus sinister, DS = Diopters Sphere, DC = Diopters cylinder, AS-OCT = anterior segment optical coherence tomography, DM = Descemet’s membrane, IOP - Intraocular pressure, GHJ = Graft-host junction, BSS = Balanced salt solution

## Introduction

DALK is the type of anterior lamellar keratoplasty in which the stroma is removed until the host’s Descemet’s membrane and replaced with donor tissue. It is usually done in patients to replace the diseased stromal tissue with a good host endothelium. Commonly, DALK is performed in keratoconus eyes and can also be used in the management of corneal degeneration and dystrophies. The big bubble technique is among the commonly used techniques to separate Descemet’s membrane from the stroma to aid easy dissection. It can sometimes prove challenging to attain a big bubble and such a scenario warrants manual dissection. This case report highlights the complications of attaining a big bubble and its subsequent management.

## Case report

A 65-year-old hypertensive male patient came to our clinic with complaints of diminution of vision and glare in the right eye for the past 3 years. His uncorrected visual acuity was 6/60 OD and 6/36 OS respectively. The right eye improved to 6/24p on correction with +1.75DS/-2.00DC at 180 and the left eye improved to 6/9 on correction with -1.25 DS/-4.00 DC at 20. Due to central spheroidal degeneration in his left eye, DALK (deep anterior lamellar keratoplasty) was performed 2 years ago.

On examination, the right eye showed Grade 3 central spheroidal degeneration associated with subepithelial fibrosis and anterior stromal scarring about 60 microns in depth, as noted in AS-OCT. Lens status was normal. The dilated fundus examination showed normal disc and retinal architecture. He was scheduled to undergo DALK on his right eye in anticipation of enhancing his visibility.

**Fig. 1:**
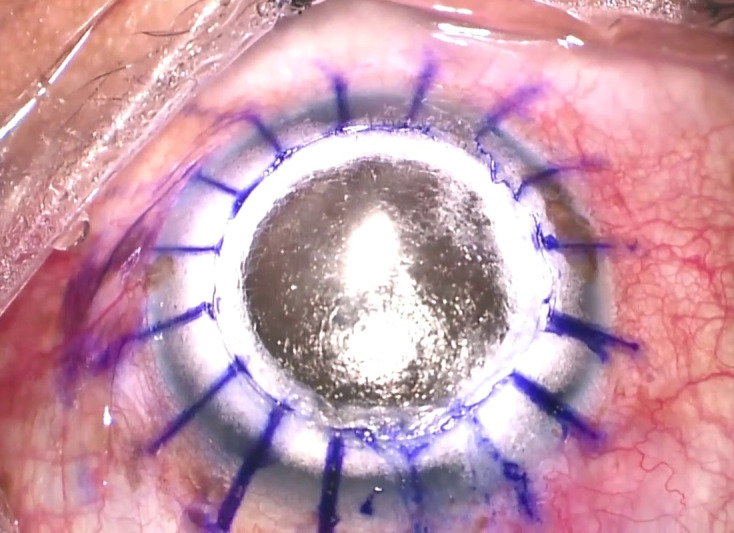
Intraoperative image showing bare stroma mimicking the surface of a type 1 bubble

Local anesthesia using 2% lignocaine with adrenaline, with gentle massage to facilitate the block, was administered in the operating room. An 8 mm trephination of the recipient bed was planned. An 8.25 mm trephine was used to punch out the donor tissue on a Teflon block without peeling the donor Descemet’s membrane (DM). A partial thickness trephination of about 400 microns was carried out. The edges were lifted using Lim’s forceps and the depth of the trephination was checked using a 15-degree blade. At that moment, a bevel-down 26-gauge needle was inserted into the cornea at the deepened groove and after reaching an appropriate distance to the corneal apex, the big bubble was tried. Failure to achieve the big bubble was noted and it was associated with stromal emphysema extending to the limbus. The big bubble was abandoned, and manual dissection was attempted. Manual dissection was carried out aiming to reach the pre-Descemet layer. Dissection ceased when the rough-looking pre-Descemet’s layer [**1**] was visualized (**Fig. 1**). 

The donor’s DM was peeled and the graft was placed over the recipient’s bed. The lamella was secured with 16 interrupted sutures.

**Fig. 2:**
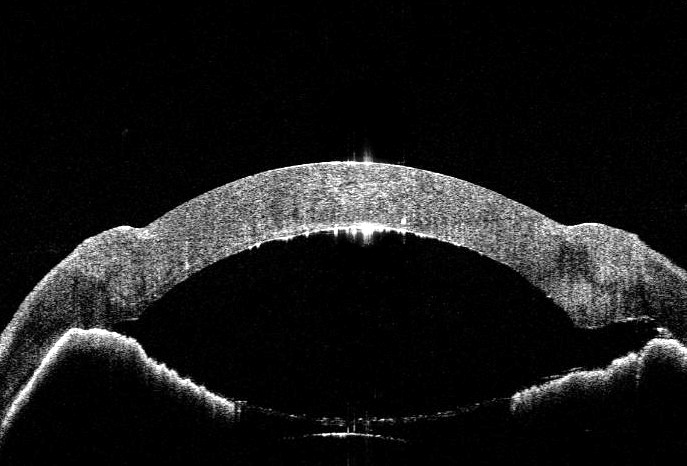
AS-OCT on postoperative day 1 - showing angle closure glaucoma by the hyperreflective host DM, causing the pupillary block due to the intact bug bubble

On postoperative day 1, the patient came with complaints of pain and swelling in the right eye. On examination, we found that the anterior chamber was shallow in the center and flat in the periphery, suspicious of an angle closure glaucoma. Applanation tonometry showed an IOP of 46 mm Hg, confirming the diagnosis. AS-OCT revealed inflated host DM, with residual stroma, which was causing a reverse pupillary block leading to an angle closure glaucoma (**Fig. 2**). 

Initial management was carried out using topical Iotim 1% (timolol) twice daily and oral acetazolamide 250 mg, 3 times a day. It was imperative to depressurize the air bubble and the patient was taken to the operating room on the second postoperative day.

In the operating room, a bevel-down 26-gauge needle was introduced from the edge of the GHJ and directed towards the corneal apex, aiming to reach the big bubble. Depressurization of the bubble was confirmed by the reflex from Descemet’s membrane and the reformation of the anterior chamber. An air bubble was introduced into the anterior chamber through the paracentesis and full air fill was maintained to ensure the complete removal of air from the interface while pressurizing the anterior chamber. After 20 minutes of balanced salt solution (BSS), air exchange was done to ensure half air fill in AC.

**Fig. 3:**
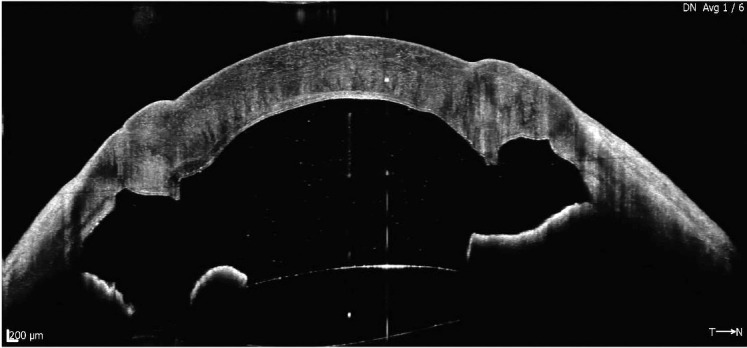
AS-OCT post deflation of big bubble showing open angles and well-attached Descemet’s membrane to the stroma

On the following day, IOP was reduced to 14 mm Hg and the anterior chamber was formed with good apposition of the DM (**Fig. 3**). AS-OCT was done, confirming good DM apposition and open angles. The patient was discharged with postoperative drops consisting of topical moxifloxacin and prednisolone. At 1 month post-op, the patient had a vision of 6/12 improving to 6/9 with pinhole.

## Results

This case report contributed to the existing volume of literature about the complications and management of deep anterior lamellar keratoplasty (DALK). It notably emphasized the rare manifestation of secondary angle closure glaucoma resulting from reverse pupillary block caused by a large bubble in a rigid cornea. The need for meticulous surgical technique and timely identification of problems was underscored to attain positive results in deep anterior lamellar keratoplasty (DALK) surgeries.

## Discussion 

We reported the first case of acute angle closure glaucoma due to a big bubble in a rigid cornea. A similar report was published by Jabbour et al. [**2**], in which DALK was done for a keratoconus patient with compromised corneal stiffness [**3**].

Increased intraocular pressure post-DALK can occur due to primary pupillary block induced by air in the anterior chamber, swollen graft, or corticosteroid response [**4**]. Steroid-induced glaucoma was highly unlikely due to the acute nature and the graft was not swollen on the first postoperative day. Air was present in the anterior chamber, but it was a partial air fill occupying 1/3 of the anterior chamber, making it unlikely to cause a pupillary block. A prompt AS-OCT (**Fig. 2**) helped clench the diagnosis of the double anterior chamber leading to secondary angle closure glaucoma due to pupillary block caused by the host’s tissue intrinsically.

Analyzing the occurrence of this event, it is quite common to mistake a thin wafer of residual stroma for a pre-Descemet’s membrane [**5**], which can sometimes bear the same rough-looking appearance as the latter, giving a false impression (**Fig. 1**). Associated emphysema can add up, making the precise identification of the Descemet’s worse. The above-mentioned issues can also occur with experienced surgeons. This predicament made it unable to realize the underlying intact big bubble, which led to reverse pupillary block and secondary angle closure glaucoma, warranting surgical intervention.

Jabbour et al. [**2**] managed the complication by removing the donor’s lamellae and continuing dissection until reaching the pre-DM layer, followed by the re-suturing of the graft to the host. We believe this was unnecessary as only 30 microns of the residual stromal bed remained and our action plan was of least intervention with favorable outcomes.

## Conclusion

We reported the first case of secondary angle closure glaucoma due to a big bubble in DALK, in a rigid cornea. It should be mentioned that stromal emphysema during a big bubble does not negate the formation of a big bubble. Prompt diagnosis and management with the least intervention are mandatory to achieve favorable outcomes in such scenarios.

**Conflict of Interest**

The authors state no conflict of interest.

**Informed Consent and Human and Animal Rights statements**

Written informed consent has been obtained from the patient included in the study. 

**Authorization for the use of human subjects**

Ethical approval: The research related to human use complies with all the relevant national regulations, and institutional policies, is in accordance with the tenets of the Helsinki Declaration, and has been approved by the institutional committee of The Eye Foundation Group of hospitals, India.

**Acknowledgments**

None.

**Sources of Funding**

None.

**Disclosures**

None.
